# Purine– and pyrimidine–triple-helix-forming oligonucleotides recognize qualitatively different target sites at the ribosomal DNA locus

**DOI:** 10.1261/rna.063800.117

**Published:** 2018-03

**Authors:** Rodrigo Maldonado, Michael Filarsky, Ingrid Grummt, Gernot Längst

**Affiliations:** 1 Biochemistry Centre Regensburg (BCR), Universität Regensburg, 93053 Regensburg, Germany; 2 Division of Molecular Biology of the Cell II, German Cancer Research Center (DKFZ), DKFZ-ZMBH Alliance, 69120 Heidelberg, Germany

**Keywords:** triplexes, triplex-forming oligos, microscale thermophoresis, ribosomal DNA

## Abstract

Triplexes are noncanonical DNA structures, which are functionally associated with regulation of gene expression through ncRNA targeting to chromatin. Based on the rules of Hoogsteen base-pairing, polypurine sequences of a duplex can potentially form triplex structures with single-stranded oligonucleotides. Prediction of triplex-forming sequences by bioinformatics analyses have revealed enrichment of potential triplex targeting sites (TTS) at regulatory elements, mainly in promoters and enhancers, suggesting a potential function of RNA–DNA triplexes in transcriptional regulation. Here, we have quantitatively evaluated the potential of different sequences of human and mouse ribosomal RNA genes (*rDNA*) to form triplexes at different salt and pH conditions. We show by biochemical and biophysical approaches that some of these predicted sequences form triplexes with high affinity, following the canonical rules for triplex formation. We further show that RNA triplex-forming oligos (TFOs) are more stable than their DNA counterpart, and point mutations strongly affect triplex formation. We further show differential sequence requirements of pyrimidine and purine TFO sequences for efficient binding, depending on the G–C content of the TTS. The unexpected sequence specificity, revealing distinct sequence requirements for purine and pyrimidine TFOs, shows that in addition to the Hoogsteen pairing rules, a sequence code and mutations have to be taken into account to predict genomic TTS.

## INTRODUCTION

Triplexes are noncanonical DNA structures comprising an additional single-stranded RNA or DNA-binding sequence specific to the major groove of double-stranded DNA. Triple helix formation has been functionally associated with transcription (Roy 1993), gene silencing (Schmitz et al. 2010), mutagenesis (Barre et al. 2000; Vasquez et al. 2000), gene conversions (Luo et al. 2000), cell proliferation (Carbone et al. 2004), and DNA double-strand breaks (Kaushik Tiwari et al. 2016). Moreover, triple helices have been reported to anchor lncRNAs to chromatin, which in turn recruit chromatin modifying complexes to regulate gene expression (Mondal et al. 2015; O'Leary et al. 2015; Postepska-Igielska et al. 2015). Despite the importance of triplexes in DNA-based processes, the knowledge about the biochemical and biophysical properties of these noncanonical DNA structures is still elusive.

**FIGURE 1.**
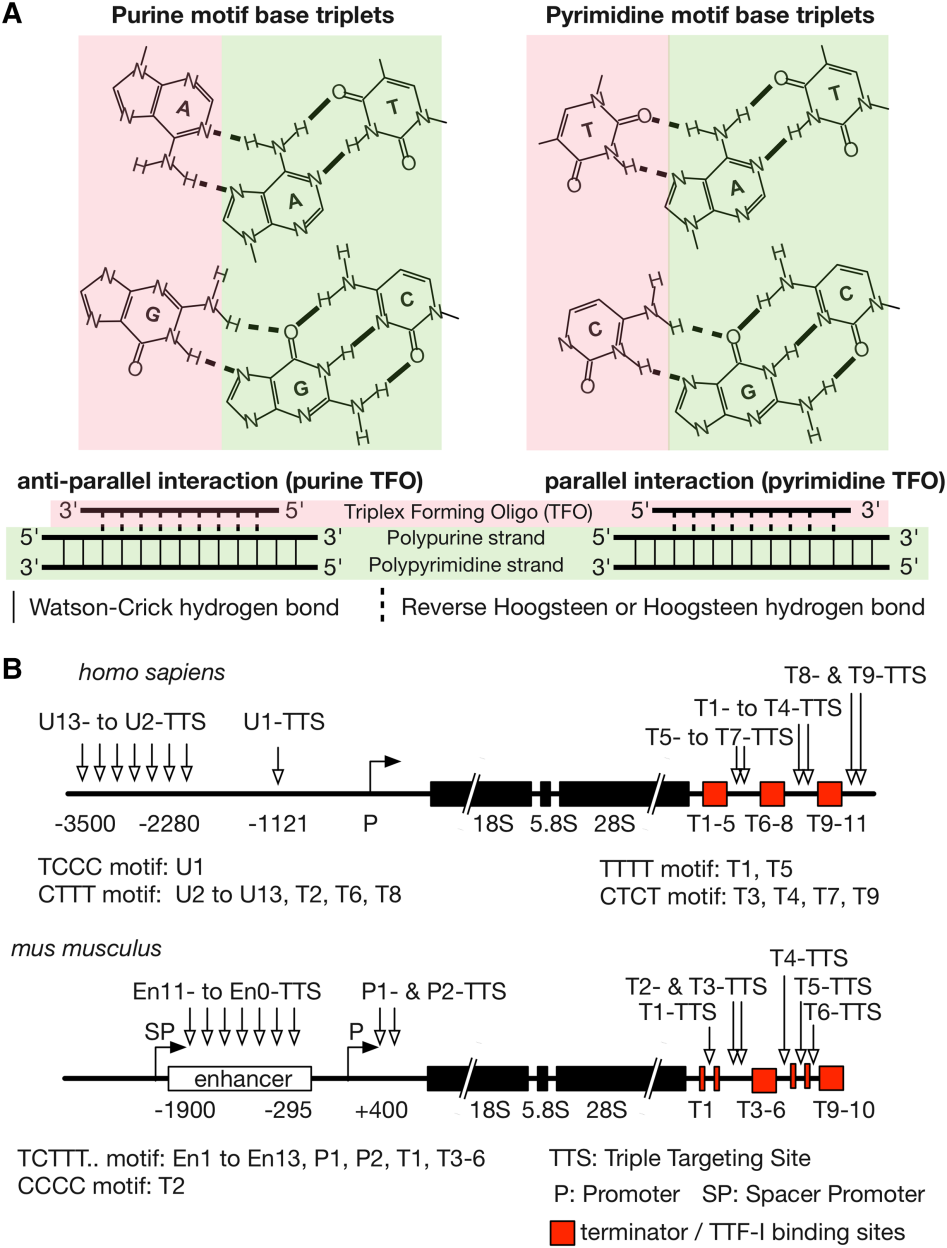
Potential TTS on the human and mouse *rDNA*. (*A*) Schematic representation of the Hoogsteen base-pairing for the purine (*left*, *up*) and pyrimidine motifs (*right*, *up*). The parallel (pyrimidine TFO) or anti-parallel (purine TFO) orientation of the third strand for the triplex formation is depicted *below* the schemes. The moieties corresponding to the third strand (TFO) and the duplex are highlighted in red and green, respectively. Watson and Crick hydrogen bonds are shown as continuous lines, while Hoogsteen hydrogen bonds as discontinuous lines. (*B*) Human and mouse *rDNA* repeat units containing putative TTS indicated by arrows. Coding sequences for ribosomal RNAs are represented with black boxes. Regulatory elements are represented as indicated in the *lower* part of the figure. Putative motifs found by Triplexator analysis on both repeat units are specified under each unit; they were named and numerated according to the regulatory element in the proximity.

Single-stranded oligonucleotides with the potential to form triple helices, termed triplex-forming oligonucleotides (TFOs), bind to the major groove of DNA and interact with polypurine sequences through Hoogsteen base-pairing (Hoogsteen 1959; Rajagopal and Feignon 1989) (Fig. 1A). Three different triplex-forming motifs have been described: (i) the T/U, C pyrimidine TFO exhibits a parallel (forward Hoogsteen) alignment of the third strand with respect to the polypurine strand orientation (Fig. 1A, right; Morgan and Wells 1968); (ii) G, A purine-rich sequences which form anti-parallel (reverse Hoogsteen) bonds with respect to the polypurine strand (Fig. 1A, left); and (iii) the G, T/U purine–pyrimidine TFO which can adopt both parallel and anti-parallel binding configurations (Beal and Dervan 1991).

As three negatively charged molecules are involved in Hoogsteen base-pairing, electrostatic repulsion forces between the phosphate groups represent the main obstacle to triplex formation. To overcome this problem, the presence of positively charged ions or larger molecules are indispensable (Felsenfeld et al. 1957). The establishment of stable, parallel Hoogsteen interactions requires the presence of divalent cations, e.g., Mg^2+^, and/or slightly acidic pH for protonation of cytosines in the TFO (Sugimoto et al. 2001; Wu et al. 2002; Chen and Chen 2011). Triplex formation follows a directional 5′ to 3′ nucleation-zipping model with respect to the polypurine strand, potentially due to the right-handed structure of the DNA (Alberti et al. 2002). A direct comparison of triplex and duplex structures reveals distortion of the DNA duplex upon the binding of the third strand, which increases the width of the major groove and makes the triplex structure more rigid than the DNA duplex (Esguerra et al. 2014).

Predictive approaches for putative triplex targeting sites (TTS), for instance with the bioinformatic software Triplexator (Buske et al. 2012), use algorithms based on the assumption that triplex formation follows the canonical binding rules of Hoogsteen base-pairing. These approaches have revealed that mammalian genomes harbor numerous TTS, which are enriched at gene promoters and regulatory elements (Goñi et al. 2004, 2006; Wu et al. 2007; Buske et al. 2012). In humans and mice, there is on average one specific TTS located ∼100–200 bp upstream of transcription start sites at every 1.3 kb within the genome (Goñi et al. 2004; Wu et al. 2007; Buske et al. 2012).

Genes encoding the ribosomal RNA (*rDNA*) are organized as long tandem repeats on the short arms of the acrocentric chromosomes (McStay and Grummt 2008; Németh and Längst 2011). The mouse genome harbors 200–400 *rDNA* repeats, each being flanked by an upstream and proximal enhancer, a terminator region downstream from the coding region (Fig. 1B) and an intergenic spacer sequence (IGS) comprising repetitive sequences (Gonzalez and Sylvester 1995; Grozdanov et al. 2003). The IGS of mouse *rDNA* contains simple repetitive sequences, mainly composed of tetranucleotides (Grozdanov et al. 2003). In contrast, the human IGS contains large polypyrimidine stretches which are exclusively located on the coding strand (Gonzalez and Sylvester 1995). In both species, these repetitive sequences are potential triple helix target sites, suggesting that RNA–DNA triplexes serve a role in nucleolar function (Gonzalez and Sylvester 1995; Grozdanov et al. 2003).

In this study, we used Triplexator-predicted TTS motifs from human and mouse *rDNA* to study the stability of triplex structures and their potential to form at physiological conditions (Fig. 1B). Triplex formation and stability was monitored by electromobility shift assays and quantified by microscale thermophoresis (MST). We show that triplexes containing an RNA TFO are more stable than DNA TFOs. Moreover, our results also reveal that triplex formation depends on both TTS and TFO sequences, being very sensitive to nucleotide mismatches. While pyrimidine TFOs preferentially form triplexes with T–A duplex-rich TTS, purine TFOs form triplex structures with G–C-rich TTS. Thus, triplex prediction according to Hoogsteen base-pairing rules is not sufficient but requires the consideration of additional structural/sequence parameters. The complementary sequence recognition code of purine and pyrimidine TFOs increases the specificity and regulatory potential of TFO containing RNA molecules.

## RESULTS

### Pyrimidine stretches within *rDNA* as potential triplex targeting sites

Mouse and human *rDNA* (BK000964 and U13369.1) were analyzed for potential triplex targeting sites (TTS) using the bioinformatic tool Triplexator (Buske et al. 2012). This software integrates the features required for Hoogsteen base-pairing to predict sequences with high potential to form triple helices. Ribosomal RNA genes from both species harbor more than 200 putative TTS, which vary in length from 15 to more than 50 bp. Interestingly, many of the potential TTS are associated with or are in close proximity to regulatory regions (Fig. 1B, sites detailed on Supplemental File S1). In mice, TTS sites are enriched at the *rDNA* enhancer (mouse enhancer: 43,375–45,144 on BK000964, motifs En11- to En0-TTS) and at the transcription termination region (mouse terminator: 13,426–14,097 on BK000964). Although the human *rDNA* enhancer is not well annotated, there is striking similarity between the location of TTS at mouse and human *rDNA*. Twelve TTS are present upstream of the human *rDNA* promoter, distributed 3.5–1.1 kb upstream of the transcription start site (motifs U13- to U1-TTS), and in the transcription termination region (T1- to T9-TTS) (Fig. 1B). In all TTS elements the purine strand is located at the noncoding strand of *rDNA*. The sequence motifs in human *rDNA* are mainly 4 bp repeats (Fig. 1B), while in mouse *rDNA*, they comprise long T stretches flanked by CT motifs (Fig. 1B; Supplemental Table S2).

### Exclusive binding of the pyrimidine TFO to the mouse *rDNA* enhancer motif

**FIGURE 2.**
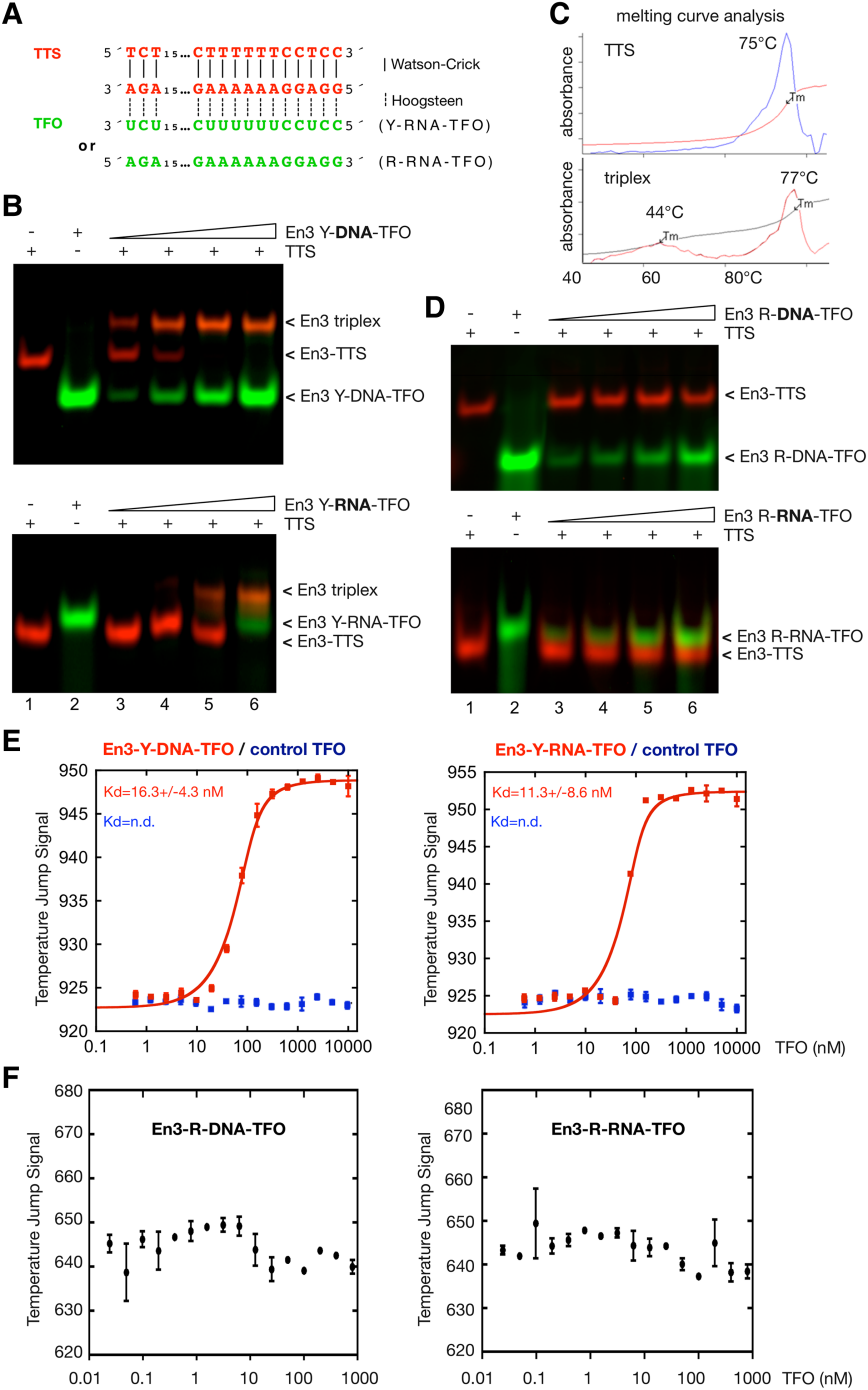
En3 TTS motifs form triplexes only with the pyrimidine TFO. (*A*) Scheme of the En3 triplet sequence formed by a pyrimidine or purine RNA TFO. The TTS and the TFO are illustrated in different colors. Watson and Crick and Hoogsteen hydrogen bonds are shown as described in Figure 1A. Titration of the FAM-labeled pyrimidine (Y) (*B*) or purine (R) (*D*) DNA–TFO (*up*) and RNA–TFO (*down*) using constant concentrations (50 nM) of the Cy5-labeled TTS (lanes *3–6*). Controls of single oligos are shown in lanes *1* (TTS, 50 nM) and *2* (TFOs, 200 nM). The TTS:TFO ratios were 1:0.5,1, 2, and 4. (*C*) Melting curve analysis of the En3 TTS alone (*up*) and forming a triple helix structure (*down*) by the addition of an equimolar concentration (500 pmol) of the En3-Y-DNA–TFO. MST analysis of the pyrimidine (*E*) and purine (*F*) En3-DNA- (*left*) and RNA–TFO (*right*) binding to the En3-TTS-Cy5 (40 nM). In *E,* the negative controls (control TFO) correspond to a GFP coding sequence and are shown in the same plots (blue squares). The graphs show the mean and standard error of three independent experiments, and dissociation constants (*K*_d_) for each TFO. (n.d.) Nondetected.

For initial analysis we chose the En3-TTS motif, which consists of the symmetric core motif TCT_(15)_CT_(6)_CCTCC and exhibits a high score for triplex formation (Fig. 2A). We assayed triplex formation by electromobility shift assays (EMSA) using fluorescently labeled En3-TTS duplex (shown in red) and increasing concentrations of a third strand comprising a fluorescently labeled pyrimidine (Y) En3-DNA or -RNA TFO (shown in green; Fig. 2B). The dual color EMSA shows that a retarded band comprising both fluorescent labels appears with increasing concentrations of pyrimidine TFO, indicating that a triple helical structure containing the TFO and the TTS has been formed. The En3-Y-DNA–TFO formed stable triplexes at a TTS:TFO ratio of 1:2 (Fig. 2B, upper panel). A similar result was obtained with the Y-RNA–TFO, which migrates slightly slower than the DNA–TTS and yields a complete bandshift at a TTS:TFO ratio of 1:4 (Fig. 2B, lower panel).

To formally prove that stable triplexes were formed under the used conditions, we performed a thermal denaturation experiment, i.e., measuring the absorbance at 260 nm over a temperature gradient from 20°C to 90°C. The melting curves show one melting point for the En3-TTS at 75°C, which represents melting of the dsDNA. If the thermal denaturation experiment was performed with an RNA–DNA triplex, there is an additional melting point at 44°C, which likely reflects the dissociation of the third strand from the DNA-duplex (Fig. 2C). While treatment with RNase H did not affect the electrophoretic mobility of the triple helical complex, the triplex disappeared after incubation with high concentrations of RNase A (Supplemental Fig. S1). These results show that the retarded band represents stable triplexes formed with the pyrimidine TFO.

However, if the En3-TTS was incubated with the respective purine TFO, predicted as high ranked TFO by Triplexator, no triplex formation was observed, regardless whether RNA or DNA purine TFOs were used (Fig. 2D). These results indicate that the Hoogsteen pairing rules are not sufficient to identify triplex-forming sequences in the genome.

### High affinity binding of the pyrimidine TFO to the mouse enhancer motif

For quantitative analysis of triplex formation, we used fluorescent microscale thermophoresis (MST), an assay that evaluates molecular interactions based on kinetic changes in a thermophoretic gradient (Supplemental Fig. S2A). The measurements require constant concentrations of the fluorescently labeled molecule and increasing concentrations of the potential binding partner. Using Cy5-labeled double-stranded En3-TTS and increasing amounts of unlabeled DNA or RNA TFOs, we observed striking changes in the thermophoretic profiles, reaching a plateau at high concentrations. In contrast, a control oligonucleotide that does not bind to En3-TTS did not change the thermophoresis profile (Supplemental Fig. S2B).

The thermophoretic profiles allowed determination of the dissociation constants (*K*_d_) for each TTS–TFO pair by analyzing changes in the temperature jump, which corresponds to the changes in quantum yield of the dye upon nucleic acid binding (Supplemental Fig. 2B). Plotting of the temperature jump signals against the TFO concentration yielded *K*_d_ values of 16.3 ± 4.3 nM for En3-Y-TFO–DNA and 11.3 ± 8.6 nM for En3-Y-TFO–RNA (Fig. 2E), reinforcing the high affinity binding of the pyrimidine TFOs. Consistent with the EMSA assays, neither the control TFO nor the purine En3 RNA– and DNA–TFO were capable of binding to the En3-TTS (Fig. 2F). Thus, the results of the MST assay reveal high binding affinity of the En3 motif for a pyrimidine-rich TFO, while the corresponding purine TFO does not recognize the predicted target site.

### Salt and pH effects on the binding affinity of TFOs

**FIGURE 3.**
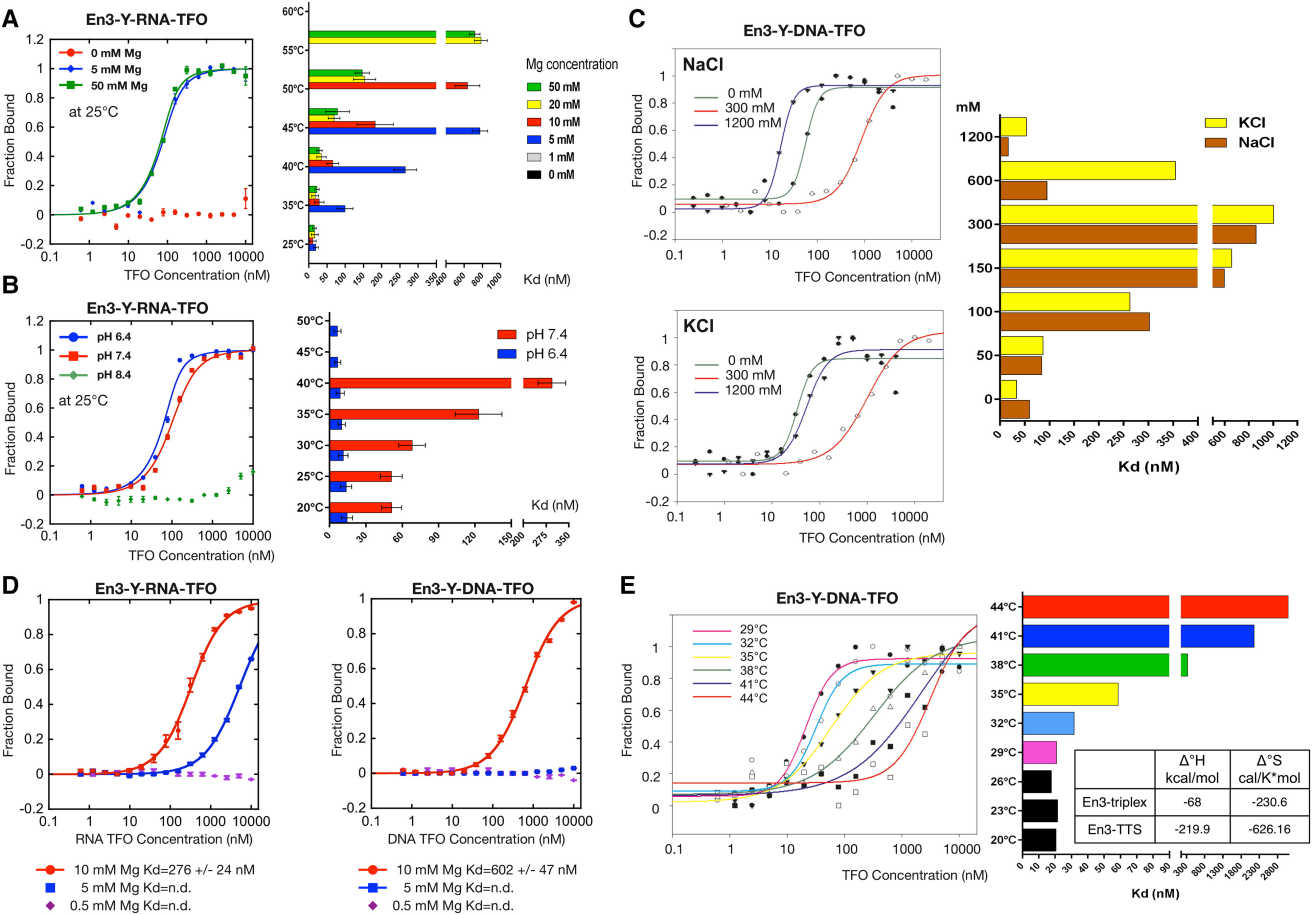
Characterization of En3 triplexes. Effect of temperature at different magnesium concentrations (*A*), and different pH values (*B*) on the En3 triplex formation. After the formation of the triplexes, the samples were incubated for another 15 min at the indicated temperatures; then analyzed by MST to obtain dissociation constants, which are shown in the bar graphs on the *right* as mean and standard error. An example of the measurements at 25°C is shown in the *left* for both cases. The graphs show the results of three independent experiments. (*C*) Triplex formation was carried out in the presence of increasing concentrations of sodium or potassium in the presence of 10 mM magnesium on MST buffer at pH 7.4. The dissociation constants at each concentration are depicted on the *right* bar graph. Examples of the MST measurements at three different monovalent cation concentrations are shown in the *left*. The complete set of measurements with all the concentrations for both cations are present in Supplemental Figure S3. (*D*) Formation of triplexes with an RNA (*left*) or DNA (*right*) TFO was performed using buffer resembling the physiological conditions of a cell, varying the concentrations of magnesium. The dissociation constant for each magnesium concentration is shown *below* each graph, which represents the results of three independent experiments. (n.d.) Nondetected. (*E*) Comparison of the dissociation constants for the En3 triplex formation at different temperatures (bar graph on the *right*). Samples were prepared on MST triplex buffer and incubated 15 min at different temperatures; prior measurements as indicated. Examples of the MST measurements are shown in the *left* side. The Δ°*H* and Δ°*S* values for the En3 triplex and the En3 TTS alone are compared in the table.

The formation and stability of triple helical structures is dependent on the presence of positive charges, magnesium being the main stabilizing ion (Felsenfeld et al. 1957; Sugimoto et al. 2001). We have quantified the thermostability of the En3 triplexes at different concentrations of magnesium, pH values, and monovalent cations. In accord with previous studies, magnesium concentrations above 5 mM are required for high affinity binding and stabilization at elevated temperatures. No triplex formation was observed in the absence and at 1 mM magnesium (Fig. 3A; Supplemental Fig. S2C). On the other hand, RNA–TFO triplexes are stable at elevated temperatures and low pH (Fig. 3B; Supplemental Fig. S2D), demonstrating that RNA–DNA triplexes are more thermostable than DNA–DNA triplexes.

The stability of triplexes is known to be affected by monovalent ions (Plum et al. 1990; Sugimoto et al. 2001). We combined increasing concentrations of physiologically relevant monovalent ions (NaCl or KCl) with constant magnesium concentrations (5 and 10 mM) (Fig. 3C; Supplemental Fig. S5, respectively). In both cases the triplexes exhibit a reduced stability for monovalent cation concentrations above 50 and below 300 mM (for measurements at 5 mM or 10 mM magnesium). At 10 mM magnesium conditions triplexes were stable at the lower and higher monovalent cation concentrations tested, exhibiting a clear concentration-dependent biphasic effect on the triplex stability. In addition, we analyzed the triplex stability at constant high NaCl concentration (600 mM), increasing the magnesium concentrations (Supplemental Fig. S6). The measurements show that Triplex stability is increased with increasing magnesium concentrations, but still in the absence of magnesium a significant binding with an *K*_d_ value of 250 nM can be observed. The data suggest that high monovalent cation concentrations can exchange the divalent magnesium to stabilize triple helices.

Next, we used a simplified physiologic buffer mimicking the ionic conditions of mammalian cells (10 mM Hepes, pH 7.4, 10 mM NaCl, 140 mM KCl, 0.05% NP40) supplemented with magnesium concentrations of 0.5 mM (free cellular concentration), 5 and 10 mM (concentration bound by proteins and nucleic acids) (Fig. 3D; Moomaw and Maguire 2008). The results indicate that stable En3 triplexes occur only at 10 mM magnesium, while at 5 mM a weak interaction between the En3-RNA–TFO to the TTS was detectable (plateau not reached), and no triplexes were formed at 0.5 mM magnesium. Noticeably, RNA–TFO triplexes exhibit a twofold better binding than the DNA–TFO at 10 mM magnesium (Fig. 3D). Still, the *K*_d_ of 276 nM for the RNA–TFO represents a binding affinity of medium strength, but indicates the requirement of additional stabilization mechanisms in the nucleus.

The dissociation constant of triplexes depends on the Gibbs free energy. To quantify the energy required for En3-TTS triplex formation, we correlated temperature-dependent changes of the *K*_d_ to changes in the enthalpy (Δ°*H*) and entropy (Δ°*S*) by using the Van't Hoff equation (Fig. 3E; Jerabek-Willemsen et al. 2014). The results show that stable En3 triplexes form between 20°C and 35°C and become increasingly unstable at higher temperatures (Fig. 3E). The Δ°*H* and Δ°*S* values of the En3 triplex (−68 kcal/mol and −230.6 cal/K*mol, respectively) are three times higher than the energy to form the En3-TTS (−219.9 kcal/mol and −626.16 cal/K*mol), demonstrating that the energy required to form the En3-triplex is approximately three times higher than the energy to form the En3-TTS.

### Point mutations dramatically affect the binding affinity of triplex sequences

**FIGURE 4.**
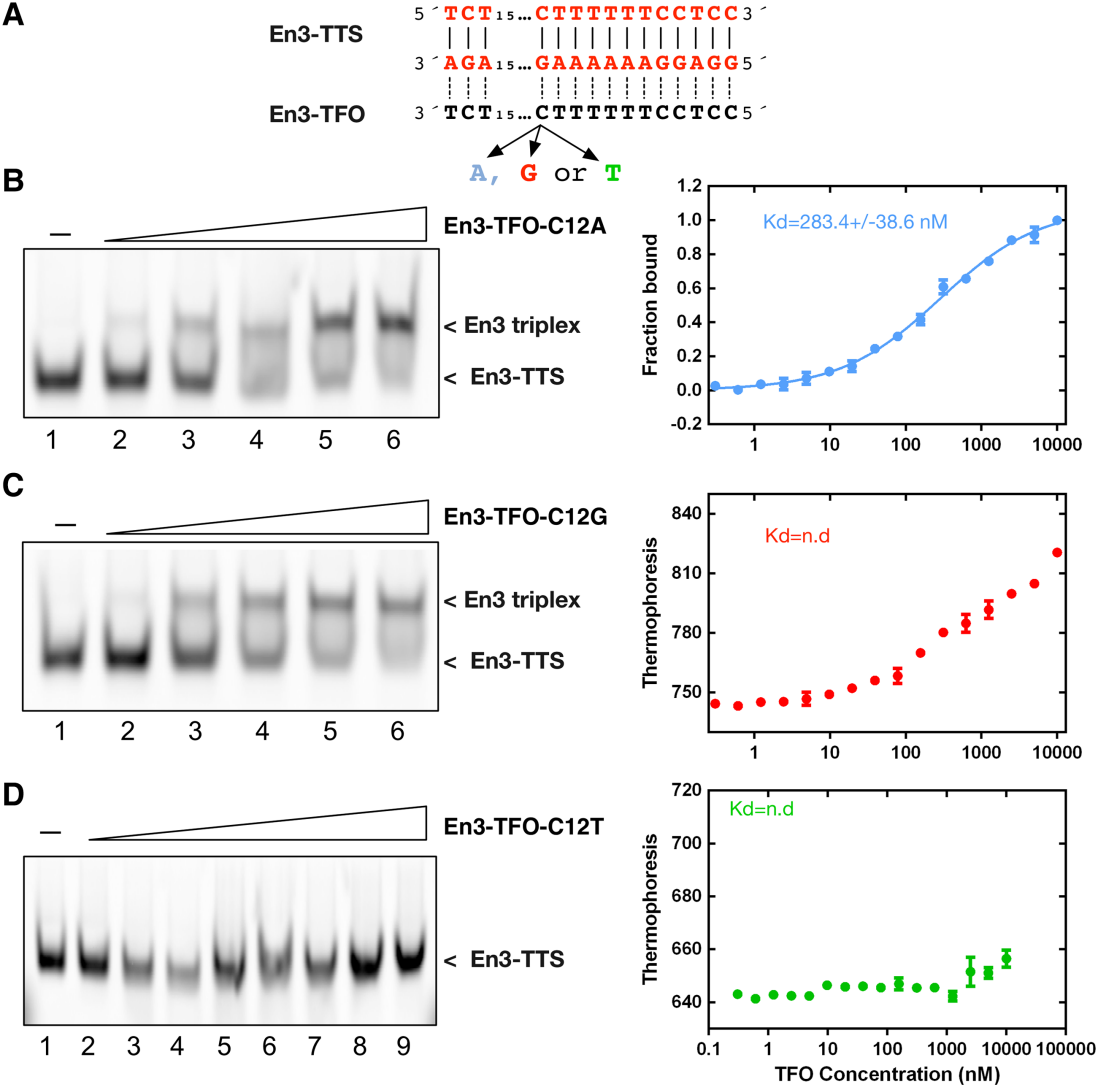
En3 triplex formation is sensitive to single mismatches in the TFO. (*A*) Representation of the single mutations generated on the C12 of the En3-Y-DNA–TFO. Titration of the nonlabeled C12A (*B*), C12G (*C*), and C12T (*D*) TFOs using constant concentrations (50 nM) of the Cy5-labeled TTS. The respective MST analyses are shown on the *right* side of each gel and represent three independent experiments. The obtained dissociation constants are specified *inside* the graphs. (n.d.) Nondetected. Lane *1* corresponds to the control in the absence of TFO. In *B* and *C* the ratios TTS:TFO were 1:0.5,1, 2, 4, and 8 (lanes *2–6*). In *D* the ratios TTS:TFO range from 1:10 to 1:200 (lanes *2–9*).

To evaluate the effect of single base pair mismatches on the En3-TFO binding to DNA, we replaced the cytosine in position 12 by adenine and guanine, respectively, and monitored triplex formation by bandshifts and MST (Fig. 4A). Both mutations reduced the binding affinity to DNA by more than one order of magnitude (Fig. 4B,C). Triplex binding lost its cooperative behavior requiring higher TFO concentrations for full triplex formation. Strikingly, replacement of the cytosine by thymidine abrogated triplex formation (Fig. 4D), demonstrating that single point mutations in the 29-bp TTS strongly impact the binding affinity and the formation of triple helices.

### A distinct binding code for purine and pyrimidine triplexes

**FIGURE 5.**
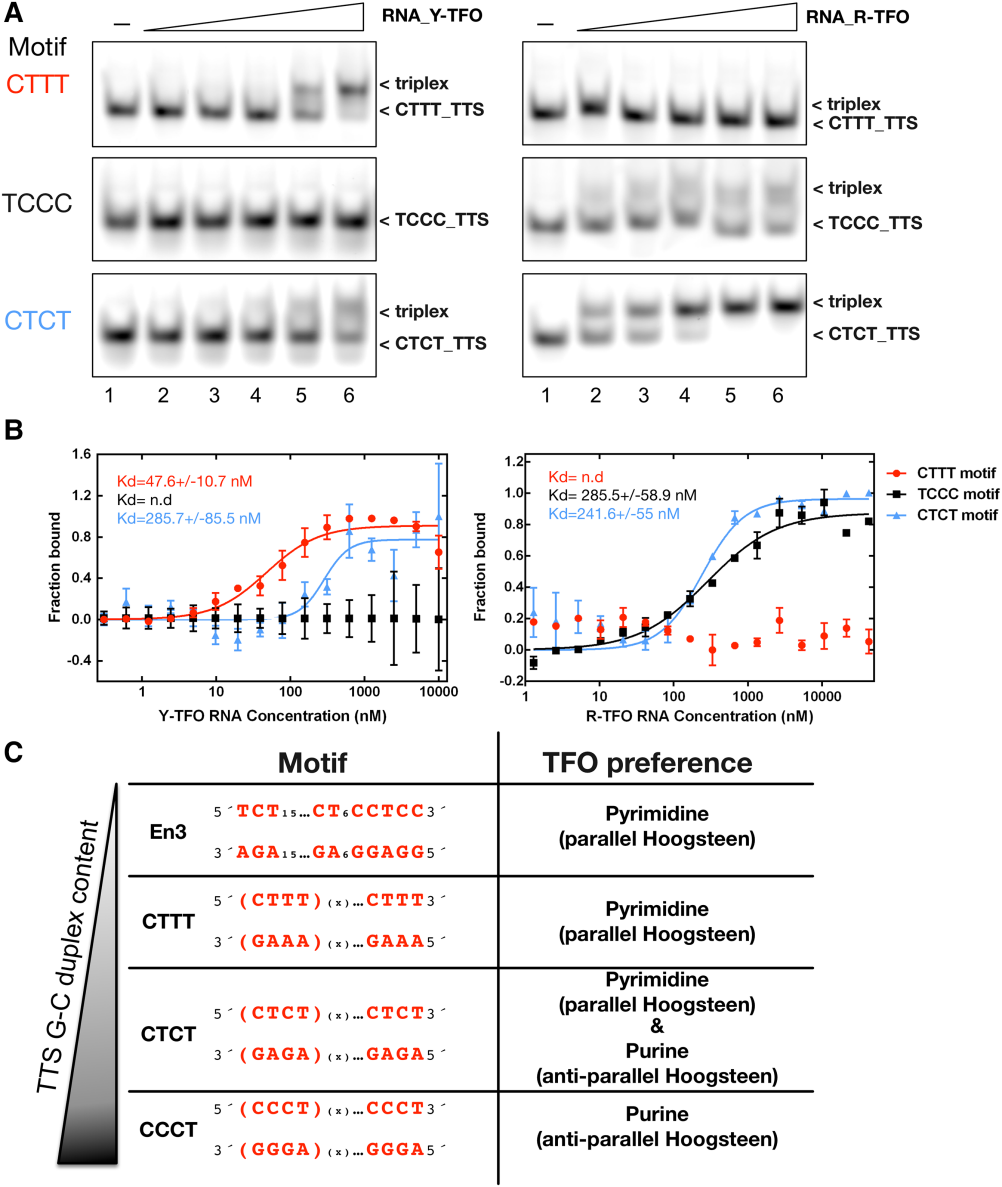
Differential triplex formation by purine and pyrimidine TFOs. (*A*) Triplex formation analysis of the different motifs found on the human rDNA with the respective pyrimidine (Y) and purine (R) -RNA–TFO by bandshift assays (*upper* panels) and (*B*) MST (*middle* panels**). In the bandshift assays, each nonlabeled RNA TFO was titrated in the presence of a constant concentration of the respective Cy5 labeled TTS (50 nM). In all the cases the ratios TTS:TFO were 1:0.5,1, 2, 4, and 8 (lanes *2–6*), and the control in the absence of TFO is shown in lane *1*. (*B*) The graphs for the MST measurements of each motif are represented by three independent experiments. The obtained dissociation constants are specified *inside* the graphs. (n.d.) Nondetected. (*C*) Overview of the TFO preference for triplex formation of the different motifs used in this study.

The triplex-forming sequences within the mouse enhancer and terminator regions present high affinity binding sites for the pyrimidine motifs. In order to study purine and pyrimidine TFO specific binding motifs, we selected the tetranucleotide sequences of the human *rDNA* (TCCC and CTTT, CTCT Figs. 1B, 5; Supplemental Fig. S4). EMSAs were performed by incubating each fluorescently labeled TTS, with increasing concentrations of the respective pyrimidine or purine RNA– (Fig. 5A) and DNA–TFOs (Supplemental Fig. S4A) at a molar ratio of 0.5:1 to 8:1 TFO:TTS. For both RNA– and DNA–TFOs we observed the same behavior, albeit with distinct binding affinities that were quantified by MST measurements (Fig. 5B; Supplemental Fig. S4B). The Y-TFOs exhibit a high affinity for the CTTT motif, full binding being achieved at a molar ratio of 4:1 and a *K*_d_ of 47.6 nM for the RNA- and 76 nM for the DNA–TFO. The binding efficiency was slightly reduced for the CTCT motif and not detectable for the TCCC motif (Fig. 5A,B; Supplemental Fig. S4A,B). Surprisingly, the R-TFOs revealed a complementary binding pattern. R-TFOs reveal medium affinity binding to the CTCT motif (241.6 nM), whereas the TCCC motif is bound with medium affinity and the CTTT motif is not bound at all (Fig. 5A,B; Supplemental Fig. S4A,B). The RNA and DNA–TFOs behave identically, however the binding affinities were slightly reduced for the RNA R-TFOs (Supplemental Fig. S4A,B). Thus, our results show that the triplex motifs have a strong sequence dependency with respect to the Y- and R-TFOs. Purine TFOs are selective for TTS rich in C–G duplexes, and pyrimidine TFOs prefer T–A duplexes (Fig. 5C).

## DISCUSSION

Putative triplex targeting sites are located in regulatory regions, mainly promoters, carrying a potential role in gene expression regulation (Goñi et al. 2004, 2006; Wu et al. 2007; Buske et al. 2012). Accordingly, it has been shown that lncRNAs are targeted to these sites, recruiting chromatin modifiers and thereby regulating gene expression (Mondal et al. 2015; O'Leary et al. 2015; Postepska-Igielska et al. 2015). Therefore, it is of key importance to understand the basics of the triplex structures formation and sequence stability.

We used the human and mouse *rDNA* units as models to study the binding and specificity of the predicted TTS, using the Triplexator software (Fig. 1B; Buske et al. 2012). Triplexator analysis gives rise to many TTS sites in *rDNA*, with most of these sequences being located in or in close proximity to regulatory elements. Here we show that not all of these predicted sequences form stable triplexes. Our results show a striking difference between purine and pyrimidine motifs. While the pyrimidine motif binds with high affinity to T-rich TTS sequences but not to C-rich TTS-sequences, the purine motif binds preferentially to sequences with alternating C–T TTS sequences and does not bind to T-rich TTS sequences (summarized in Fig. 5C). We observed this for the tetranucleotide-repeats of human *rDNA* but also with the nonrepetitive mouse enhancer sequence En3. In addition, previous reports have described that TFOs containing only T residues are highly unstable when compared to TFOs comprising different ratios of T and C (James et al. 2003). The exclusive binding of the purine TFO to the TCCC motif was certainly unexpected, because previous reports showed that the CCT motif (15mer) was able to form triplexes with the respective pyrimidine TFO (James et al. 2003). In addition, we observe that the CTCT motif could efficiently form triplexes with both types of TFOs (Fig. 5A; Supplemental Fig. S4A).

Our results imply that RNA sequences are highly sequence specific toward TTS sites, since the kind of TFO-sequence (R or Y), the target sequence composition and the perfect sequence match creates a highly selective TFO–TTS recognition motif. Large differences in the stabilities (Gibbs free energy) were observed by comparing triplexes and the corresponding TTS. Thus, in addition to the Y/R TFO selectivity, the energy needed to stabilize these structures is a key parameter to decide between a pyrimidine– or a purine–TFO for a specific TTS. Accordingly, single mismatches strongly perturb the TFO–TTS interaction (Moser and Dervan 1987; Mergny et al. 1991; Roberts and Crothers 1991; Alberti et al. 2002). One C to A or a C to G mismatch in a 29-bp long TTS site reduced the binding affinity by an order of magnitude, while a C to T mismatch completely inhibited the formation of a stable triplex (Fig. 4). The En3-triplex contains stretches of T•A–T triplex bases interrupted by a C•G–C triplet (Fig. 4A). This single base “interruption” stabilizes the triplex (Giovannangéli et al. 1992) and is responsible for the high affinity of the En3-TFO. On the other hand, replacement of C by T on the TFO explains destabilization of the En3-triplex formation by a single base mismatch.

Using the quantitative microscale thermophoresis method (MST), we performed a thorough analysis of TFO–TTS binding affinities at different salt, pH and temperature conditions. We observed similar and low *K*_d_ values (∼10–15 nM) for both DNA and RNA pyrimidine En3-TFOs, and a highly cooperative binding curve (Fig. 2E). We established as well the strong dependency of these high affinity complexes on the presence of Mg^2+^. At limiting magnesium concentrations the RNA–TFO exhibits higher thermostability than the DNA–TFO (Fig. 3A). The increased thermostability of the RNA–TFO was also observed at different pH values (pH values 6.4 and 7.4, Fig. 3B), and under buffer conditions resembling the cellular environment (Fig. 3D). These results are consistent with previous studies showing that dsDNA+ssRNA triplets are more stable than dsDNA+ssDNA triplexes, and suggest that the stability of RNA–TFO triplexes are in the range of specific DNA–protein interactions (Roberts and Crothers 1992; Escudé et al. 1993).

The magnesium dependency for the triplex formation stabilization relies on the direct contact of this cation with the dsDNA. This divalent cation interacts both grooves of a duplex, but specifically, Mg^2+^ bridges the phosphate groups at the top of the minor groove narrowing it (Hud and Polak 2001). Thus, the interaction of Mg^2+^ with the unoccupied minor groove of a triple helix structure would aid to stabilize this structure. Regarding the differences observed at different pH values, they could be explained by the pyrimidine nature of the TFO used for the En3 triplexes, which contains six cytosine residues. The parallel binding of a pyrimidine TFO depends on the cytosine protonation, that occurs on both neutral and acidic conditions, in order to stabilize the Hoogsteen interactions (Hüsler and Klump 1995; Lavelle and Fresco 1995).

The analysis of the effect of monovalent cations on triplex formation has revealed that both Na^+^ and K^+^ cations stabilize En3 triplexes at low (up to 50 mM) and high (above 300 mM) concentrations, while binding affinities are reduced at physiological concentrations (Fig. 3C; Supplemental Fig. S5). Focusing on the effects of Na^+^ in the presence of Mg^2+^, it is known that both ions interact with the triple-oligonucleotide complex but in a different manner. Na^+^ has been described not to bind to specific positions, while Mg^+2^ mainly interacts with the phosphate groups at the top of the minor groove (Hud and Polak 2001; Sugimoto et al. 2001). We observed that high sodium concentrations (600 mM), even in the absence of Mg^2+^ facilitates the triplex formation. However, at the same conditions, high levels of triplex stability are reached only in the presence of the divalent cation (Supplemental Fig. S6). Therefore, the observed biphasic effect and the stabilization of triplexes by Mg^2+^ at high Na^+^ concentration, may represent a competition between the mono- and divalent cations as previously described (Sugimoto et al. 2001).

To mimic cellular conditions we analyze the triplex formation on a buffer containing 150 mM of monovalent cations (10 mM Na^+^ and 140 mM K^+^) and different magnesium concentrations. Our results showed that triplexes are preferentially stabilized at 10 mM Mg, with *K*_d_ values (276 nM for the RNA–TFO and 602 for the DNA–TFO) representing a binding affinity of medium strength (Fig. 3D). These results are in agreement with the observed effects of monovalent ions on triplex formation (Fig. 3C). In both cases, for the DNA–TFO, we obtained similar *K*_d_ values (∼600 nM) at 150 mM ionic strength and 10 mM Mg^2+^. This indicates that at physiological ion concentrations, efficient triplex formation requires the presence of additional stabilizing components, like for example proteins that facilitate the accommodation of a single-stranded oligonucleotide in the major groove of a duplex. This could be the case for DNMT3 that binds, and probably stabilizes, a triplex structure on the mouse *rDNA* (Schmitz et al. 2010). Moreover, we speculate that the UBF protein, binding to the rDNA enhancer with its triplex motifs, could be a triplex stabilizing protein. UBF is a major activator of rDNA transcription and exclusively bound to the enhancer of active *rDNA* copies (Jantzen et al. 1990). UBF belongs to the group of high mobility group box (HMG-box) containing proteins that were shown to bind to and to stabilize triple helices (Suda et al. 1996).

Chromatin-associated RNAs are enriched in GA- and GT-TFO sequences (Buske et al. 2012). Nevertheless, this tendency cannot be generalized, as there is a clear enrichment of TC and GT sequences in nucleolar chromatin-associated RNA (Caudron-Herger and Rippe 2012). Thus, the TFOs studied here represent potential target sites in mammalian cells. The human rDNA motifs (TCCC, CTTT, and CTCT) correspond to repetitive sequences, which form mirror TFOs that could target other genomic regions or their origin (*cis*- and *trans*-targeting) (Jain et al. 2008). In the case of the mouse En3 motif, this sequence is asymmetric and transcription of the enhancer region will not allow *cis*-targeting. However, a simple BLAST search (Basic Local Alignment Search Tool) (Altschul et al. 1997) identified different transcripts containing almost the complete En3 TFO, most of them lacking only two or three bases with a 100% identity for the rest of the sequence (Supplemental Table 3), indicating that a *trans*-targeting mechanism could apply to regulate the mouse *rDNA* transcription.

Predictive tools to identify TTS and TFOs are very useful to investigate potential triplexes in vivo. However, these tools use algorithms based on the canonical Hoogsteen base-pairing rules to justify the prediction of sequences, which we show is not sufficient. In this study we have shown that triplex formation is highly sequence specific, where pyrimidine TFOs will preferentially form triplexes with T–A duplex rich TTS's, and purine TFOs with G–C duplex rich TTS sequences. Therefore, additional experimental rules or further computer modeling parameters, like the impact of the energy needed to distort a specific TTS with the proper TFO to form a triplex, must be taken into account.

## MATERIALS AND METHODS

### Determination of putative TTS on mouse and human rDNA

Potential triplex targeting sites (TTS) on the mouse (GenBank: BK000964) and human *rDNA* (GenBank: U13369.1) were identified with the Triplexator software package (Buske et al. 2012). The results of both analyses performed with permissive parameters in terms of the percentage of guanines (lower limit of 0%) and the length of the sequences. The error rate was 5% and the consecutive error was set as 1 (maximum permitted is 3). The analyses revealed several potential TTS for the human and mouse *rDNA*, being located close to regulatory regions (Fig. 1B; Supplemental File S1). The sequences of mouse *rDNA* that are specifically located at the enhancer are listed in Supplemental Table S2.

### Electrophoretic mobility shift assays (EMSA)

TTS (dsDNA) sequences were prepared by mixing the forward and reverse strands at equimolar ratios in oligo annealing buffer (20 mM Tris–HCl [pH 7.4], 2 mM MgCl_2_, 50 mM NaCl). The mixture was heated for 5 min at 95°C and then slowly cooled down to RT. To examine triplex formation, 50 nM TTS were incubated with increasing concentrations of the TFO (ssDNA or ssRNA) and incubated in triplex annealing buffer (40 nM Tris-acetate [pH 7.4], 10 mM Mg-acetate) for 15 min at 25°C. Triplex formation was monitored by electrophoretic mobility shift assays (EMSAs) on 15% polyacrylamide gels in 40 mM Tris-Acetate [pH 7.4], 10 mM Mg-acetate at 15 Volt/cm. TTS and/or TFO molecules were fluorescently labeled with Cy3, Cy5 or FAM (as indicated in the individual experiments) and the gels were analyzed with a fluorescence reader (Typhoon FLA 9500 GE Healthcare Life Sciences). All sequences are listed in Supplemental Table S1.

### Melting temperature analysis

Measurements of the melting curves were performed on a Cary 100 Bio UV-Visible spectrophotometer. The temperature was increased from 25°C to 100°C at a rate of 2°C/min and the absorbance of the samples (200 µL) was measured at 260 nm. The melting temperature analysis was performed at equimolar ratios of mTTS-En3 and mTFO-En3 DNA (500 pmol). The melting temperature plot was generated by the Cary Win UV/Thermal software.

### Microscale thermophoresis (MST)

The MST method allows the quantitative measurement of molecular interactions in solution based on the physical effect called thermophoresis. The kinetics of molecular movement depends on parameters like size, charge and hydration shell. The interaction of two molecules will change these parameters and affect the kinetic behavior of the complex (Supplemental Fig. S2A). MST measurements were performed with a constant concentration of the fluorescently labeled triplex target site (TTS; 10–40 nM), adding increasing concentrations of TFO as a serial dilution series (0.5 nM–10 µM). Reactions were performed in MST triplex buffer (40 mM Tris-acetate [pH 7.4], 10 mM Mg-acetate, 0.05% NP40), incubated 15 min at 25°C and taken up into glass capillaries for measurement (Monolith NT.115, NanoTemper, Germany) (Supplemental Fig. S2B). Data sets were plotted and the dissociation constant (*K*_d_) was calculated according to the law of mass action (Baaske et al. 2010).

Triplex stability and the corresponding effect of buffer composition were analyzed by heat controlled MST reactions using MST buffers with varying Mg^2+^ concentrations (0–10 mM: Fig. 3A, and Supplemental Fig. S2C; 0–100 mM: Supplemental Fig. S6), pH ([pH 6.4/7.4/8.4]; Fig. 3B and Supplemental Fig. S2D) and monovalent ions between 0 and 1200 mM (Fig. 3C; Supplemental Fig. S5). The analysis of triplex formation resembling the physiologic conditions was performed in MST triplex cell buffer (10 mM Hepes, pH 7.4, 10 mM NaCl, 140 mM KCl, 0/5/10 mM MgCl_2_, 0.05% NP40) (Fig. 3D).

## SUPPLEMENTAL MATERIAL

Supplemental material is available for this article.

## Supplementary Material

Supplemental Material
