# Supplementary material for: Purine– and pyrimidine–triple-helix-forming oligonucleotides recognize qualitatively different target sites at the ribosomal DNA locus
Source: RNA. 2018 Mar;24(3):371–80. doi: 10.1261/rna.063800.117 (PMC5824356; doi:10.1261/rna.063800.117)
Supplement: Supplemental Material [file supp_24_3_371__index.html]

Purine– and pyrimidine–triple-helix-forming oligonucleotides recognize qualitatively different target sites at the ribosomal DNA locus — Supplemental Material 

# Purine– and pyrimidine–triple-helix-forming oligonucleotides recognize qualitatively different target sites at the ribosomal DNA locus

## Supplemental Material

- Supplemental\_Fig\_S1.tiff
- Supplemental\_Fig\_S2.tiff
- Supplemental\_Fig\_S3.tiff
- Supplemental\_Fig\_S4.tiff
- Supplemental\_Fig\_S5.tiff
- Supplemental\_Fig\_S6.tiff
- Supplemental\_File\_S1.xlsx
- Supplemental\_Table\_S1.tiff
- Supplemental\_Table\_S2.tiff
- Supplemental\_Table\_S3.tiff
