# Supplementary figures and images for: Purine– and pyrimidine–triple-helix-forming oligonucleotides recognize qualitatively different target sites at the ribosomal DNA locus
Source: RNA. 2018 Mar;24(3):371–80. doi: 10.1261/rna.063800.117 (PMC5824356; doi:10.1261/rna.063800.117)

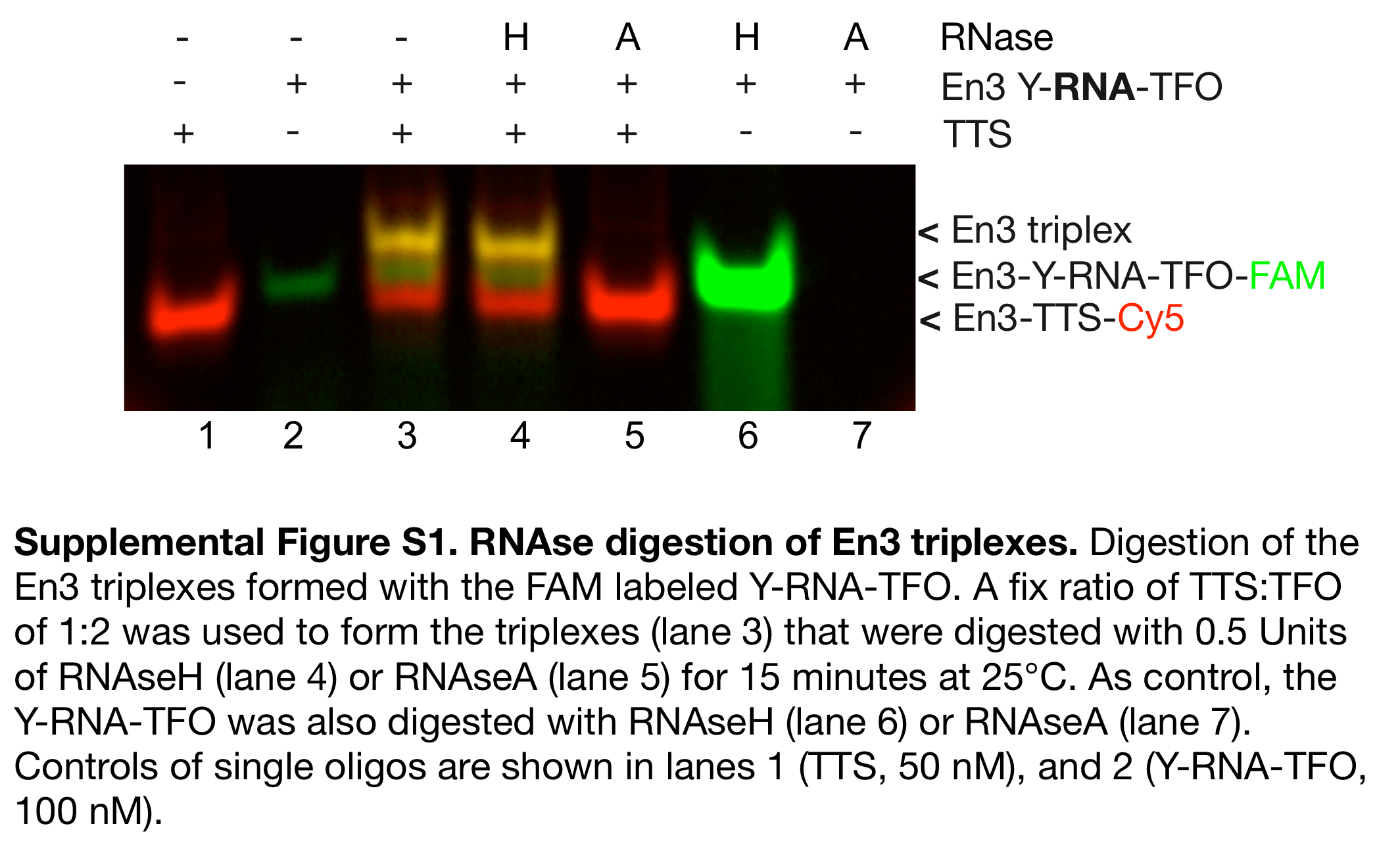

Supplement: Supplemental Material [file supp_063800.117_Supplemental_Fig_S1.tif]

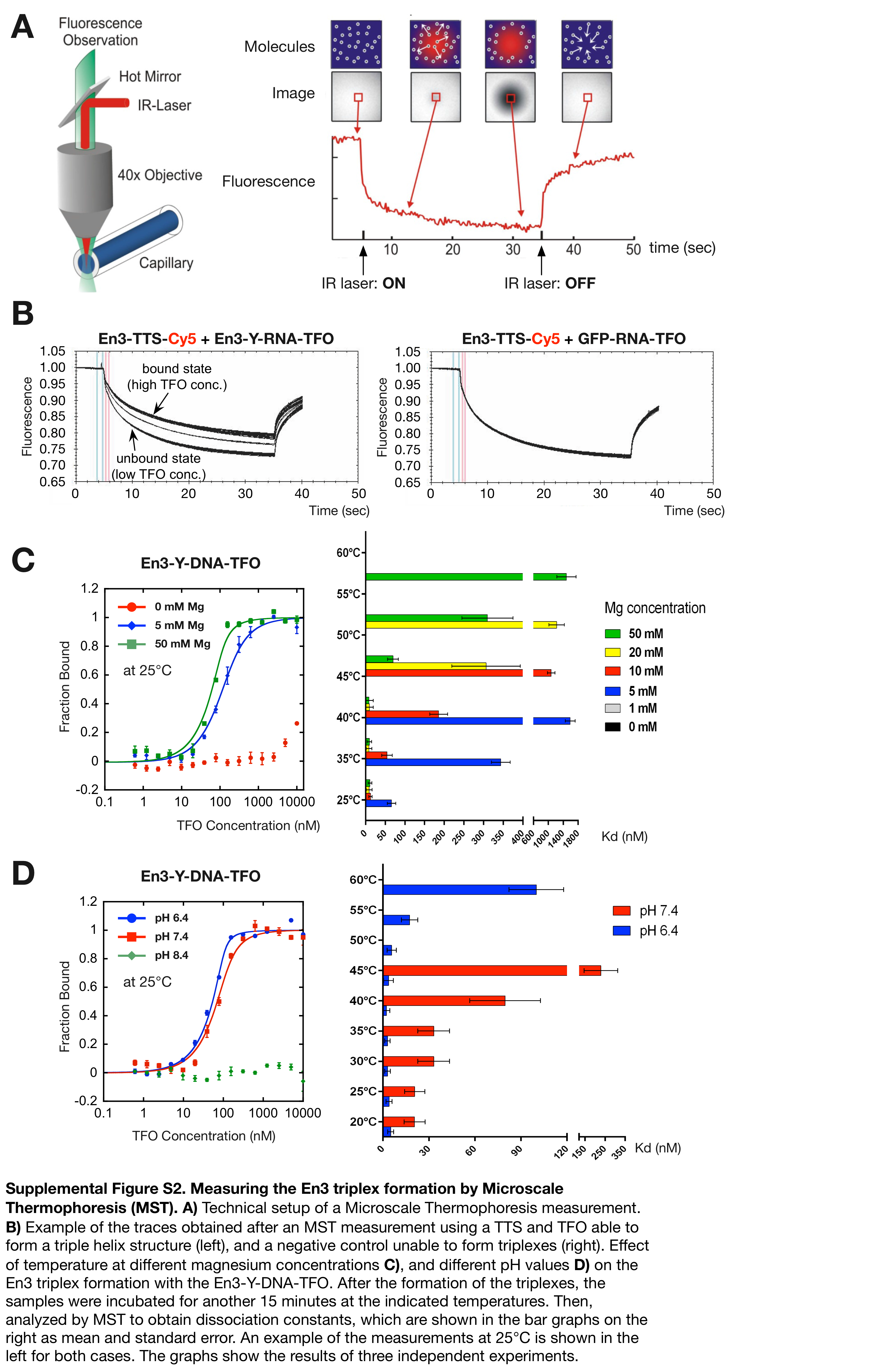

Supplement: Supplemental Material [file supp_063800.117_Supplemental_Fig_S2.tif]

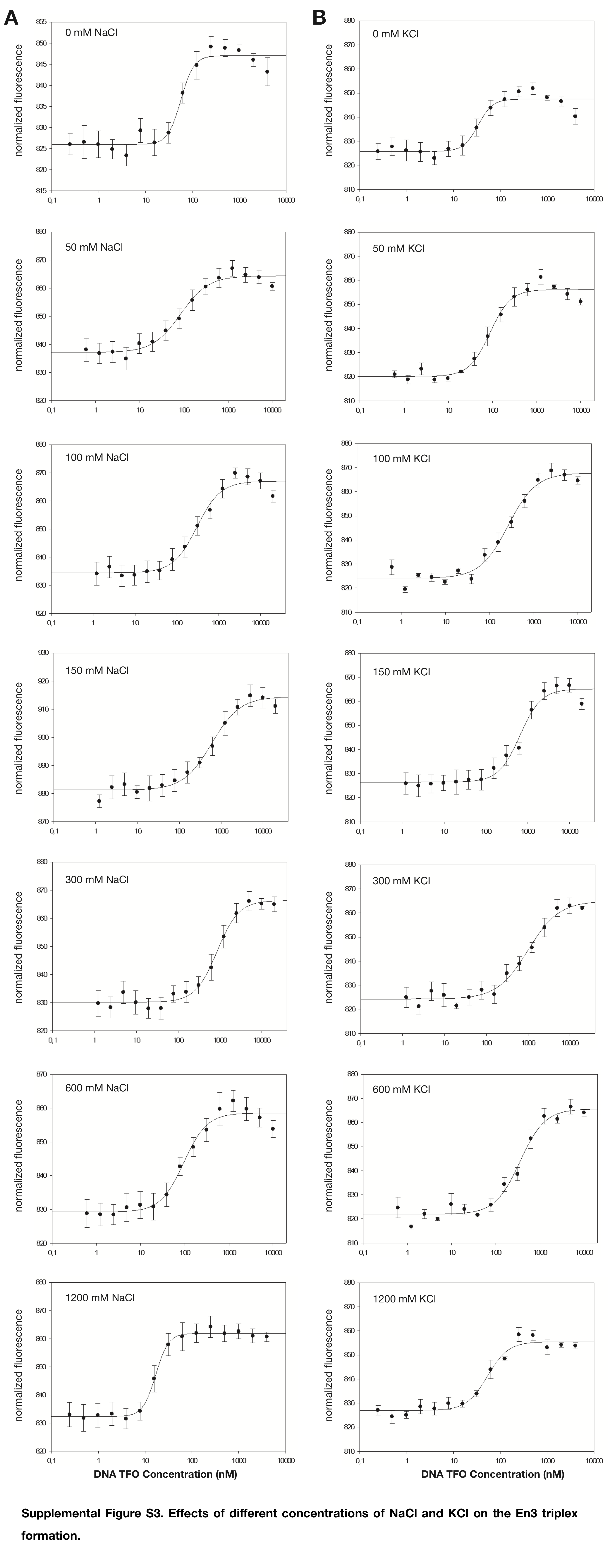

Supplement: Supplemental Material [file supp_063800.117_Supplemental_Fig_S3.tif]

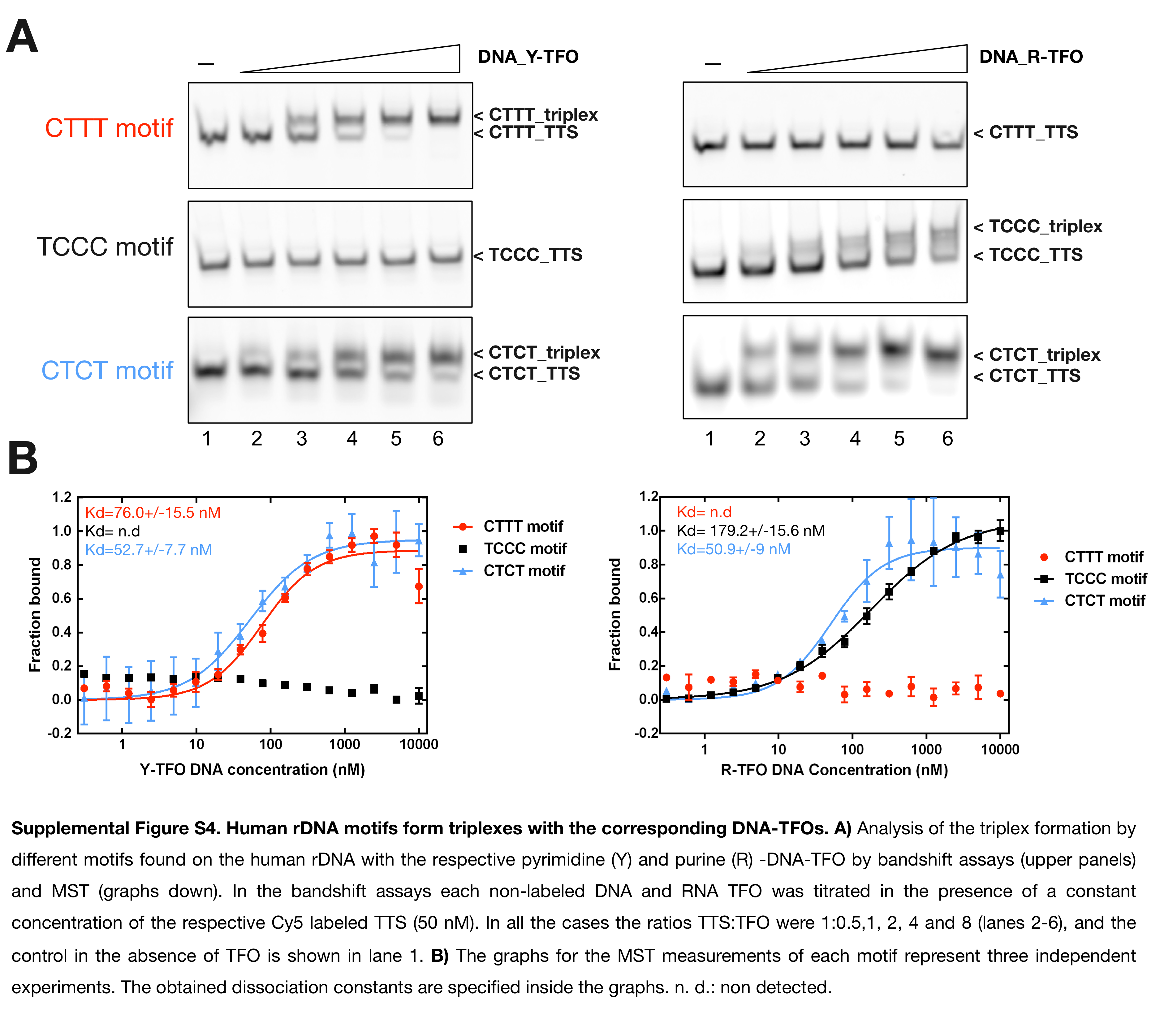

Supplement: Supplemental Material [file supp_063800.117_Supplemental_Fig_S4.tif]

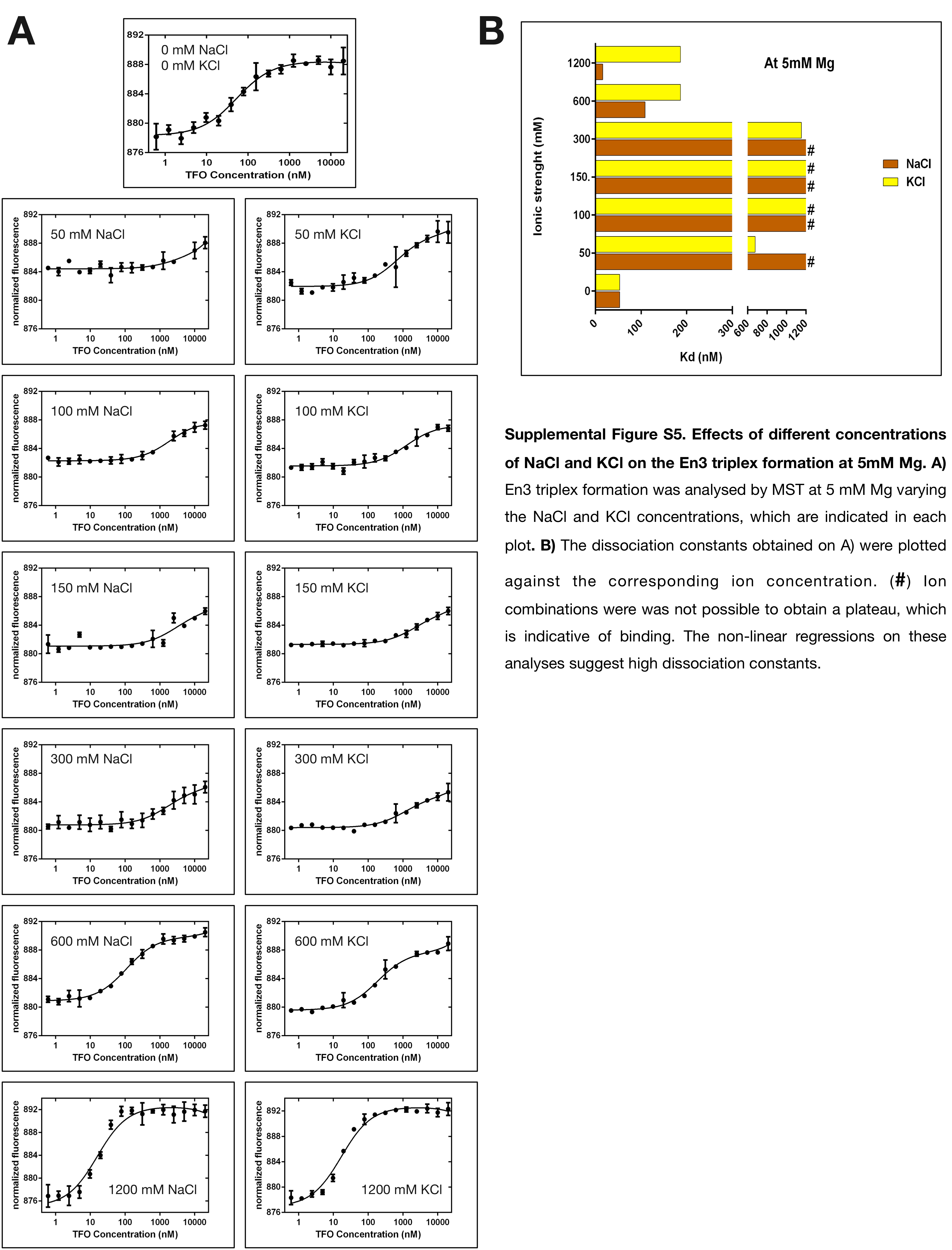

Supplement: Supplemental Material [file supp_063800.117_Supplemental_Fig_S5.tif]

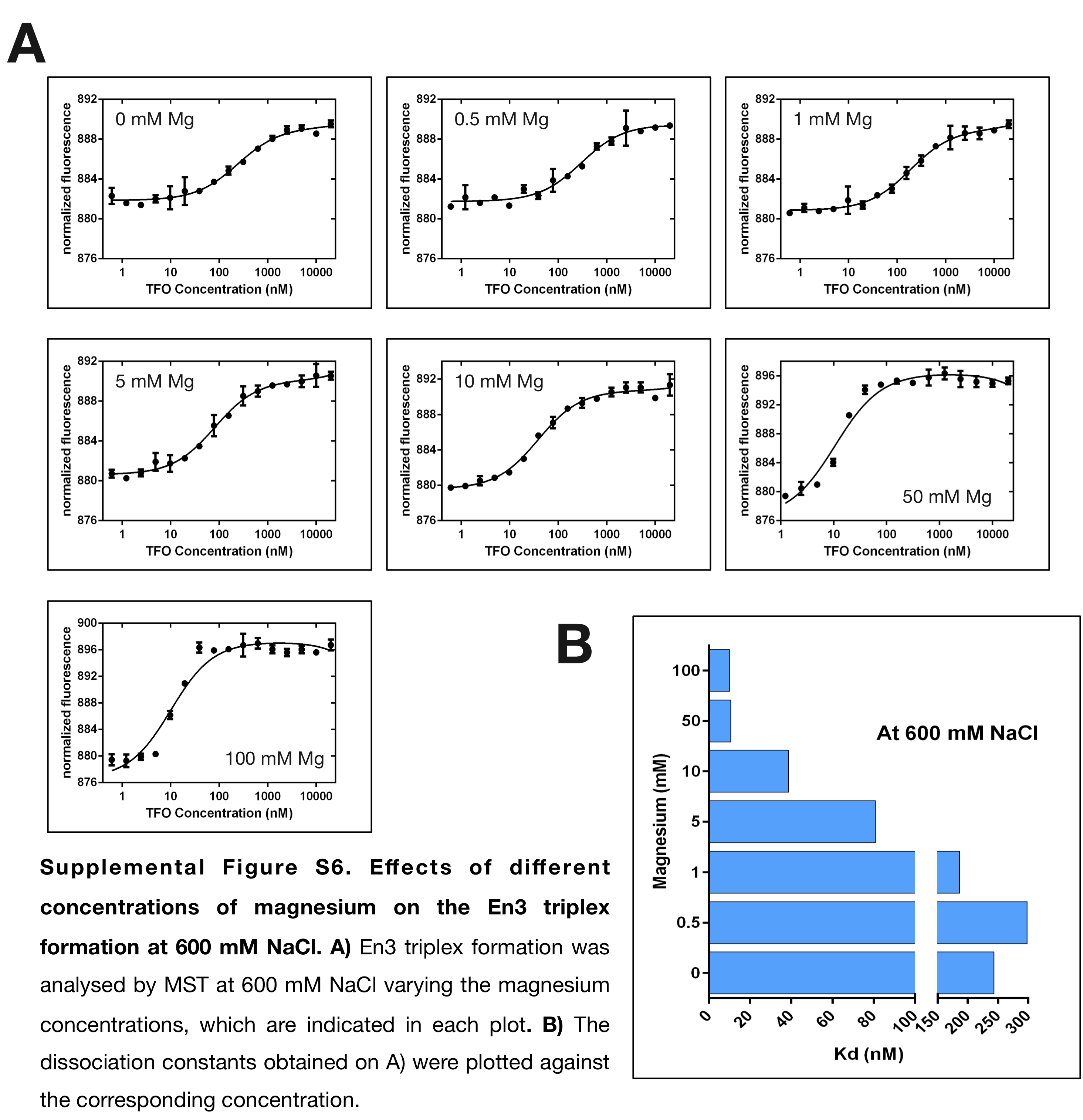

Supplement: Supplemental Material [file supp_063800.117_Supplemental_Fig_S6.tif]

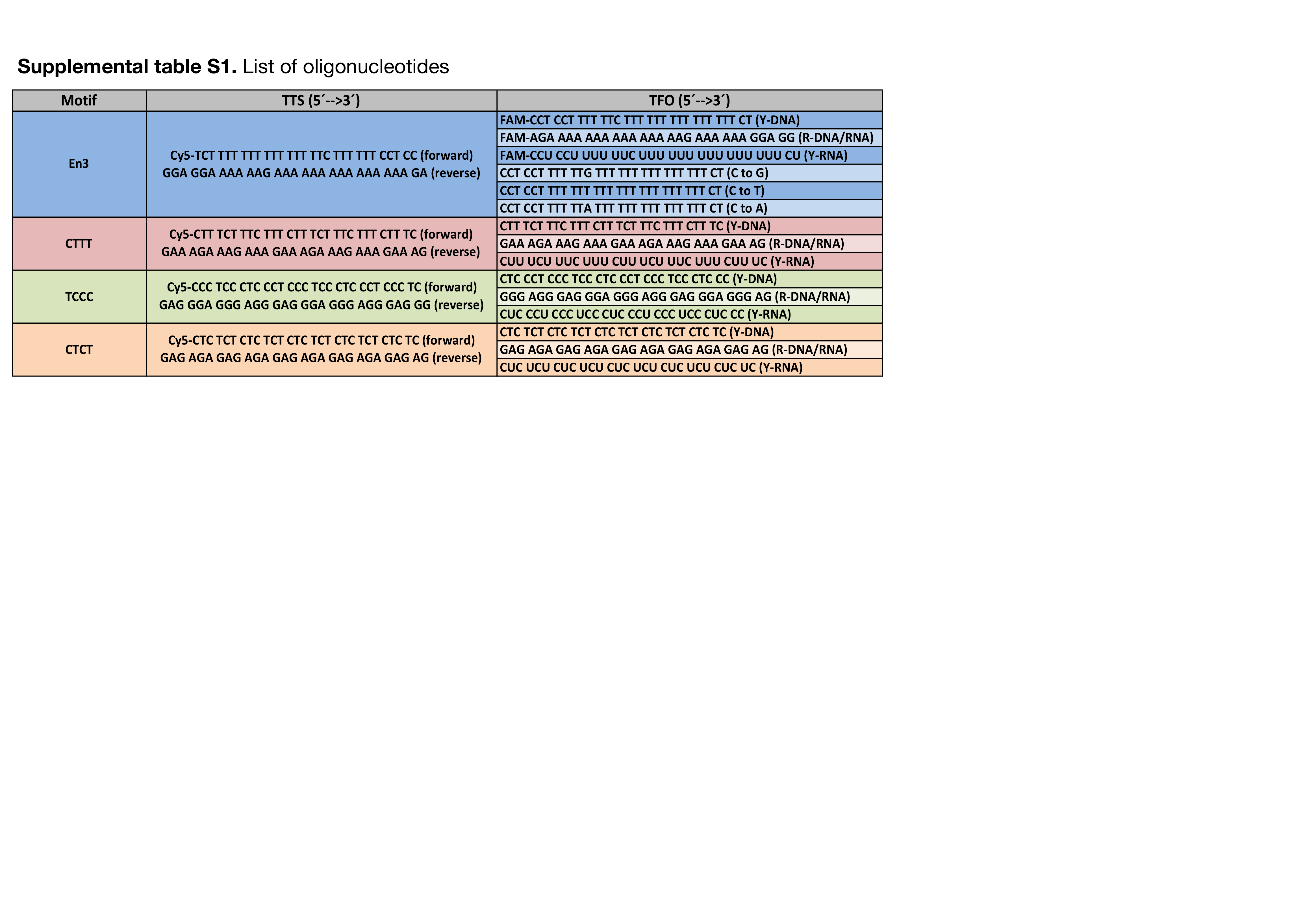

Supplement: Supplemental Material [file supp_063800.117_Supplemental_Table_S1.tif]

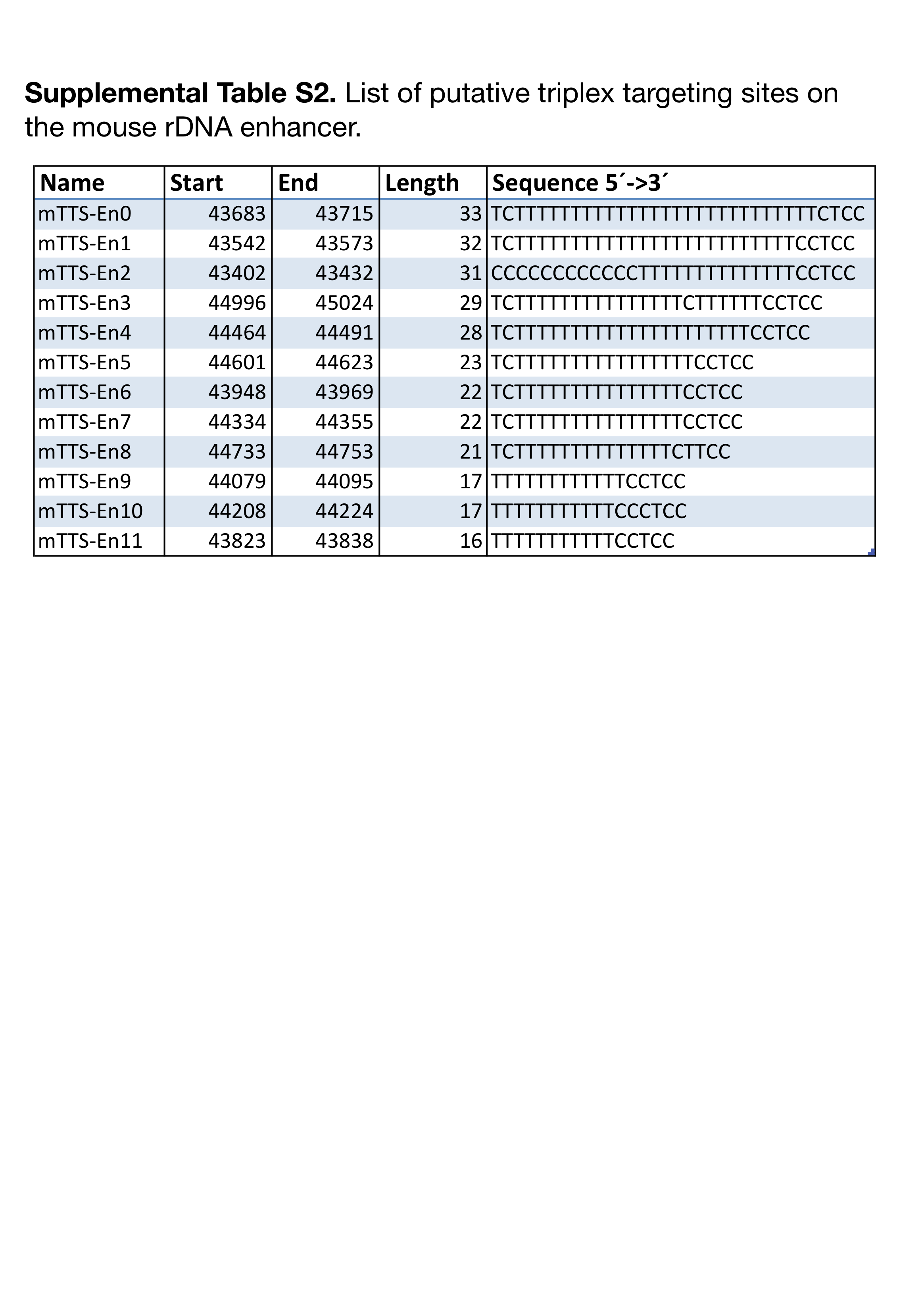

Supplement: Supplemental Material [file supp_063800.117_Supplemental_Table_S2.tif]

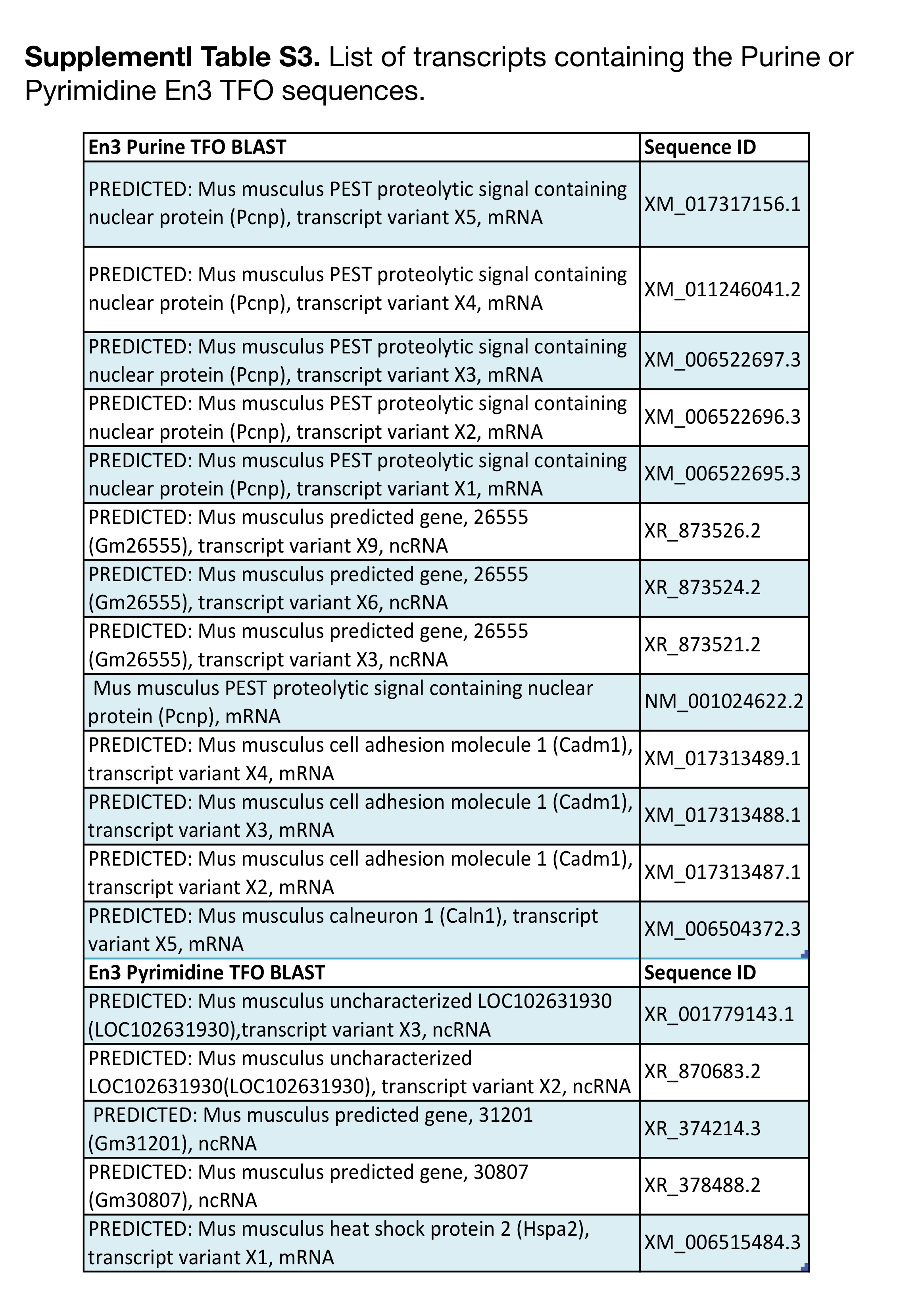

Supplement: Supplemental Material [file supp_063800.117_Supplemental_Table_S3.tif]
